# Establishing a new species group of *Pseudopoda* Jäger, 2000 with the description of two new species (Araneae, Sparassidae)

**DOI:** 10.3897/zookeys.879.35110

**Published:** 2019-10-09

**Authors:** He Zhang, Peter Jäger, Jie Liu

**Affiliations:** 1 The State Key Laboratory of Biocatalysis and Enzyme Engineering of China, College of Life Sciences, Hubei University, Wuhan 430062, Hubei, China Hubei University Wuhan China; 2 Arachnology, Senckenberg Research Institute, Senckenberganlage 25, 60325 Frankfurt am Main, Germany Senckenberg Research Institute Frankfurt am Main Germany; 3 School of Nuclear Technology and Chemistry & Biology, Hubei University of Science and Technology, Xianning 437100, Hubei, China Hubei University of Science and Technology Hubei China

**Keywords:** Biodiversity, systematics, taxonomy, huntsman spiders, China

## Abstract

The huntsman spider genus *Pseudopoda* Jäger, 2000 contains 140 species worldwide, of which 61 have been described from China. In this paper, this knowledge is increased by the description of two new species from Yunnan Province in China. These new species, *P. physematosa***sp. nov.** (♀) and *P. semilunata***sp. nov.** (♂♀), are treated with five previously described ones, *P. bibulba* Xu & Yin, 2000 (♂♀), *P. signata* Jäger, 2001 (♂♀), *P. wu* Jäger, Li & Krehenwinkel, 2015 (♂♀), *P. yinae* Jäger & Vedel, 2007 (♂), and *P. yunnanensis* Yang & Hu, 2001 (♂♀), as the newly defined *Pseudopoda signata* species group. The *P. signata* group can be distinguished from other groups within *Pseudopoda* by the male palps with long, slightly broad, S-shaped embolus, small but distinct tegular apophysis, pronounced dRTA and reduced vRTA, and by the female with V-shaped or W-shaped anterior margins of lateral lobes, membranous and wide first winding, long and strongly curved SIDS (sclerotised internal duct system), the latter mostly covered by the first winding. The monophyly of this group is also supported by molecular phylogenetic results mainly based on Chinese *Pseudopoda* species. In addition, photographs of *P. bibulba* (♂♀), *P. signata* (♂♀), and *P. yunnanensis* (♂♀) are provided. *P. bibulba* is newly recorded from Guizhou Province and *P. signata* is newly recorded from Yunnan Province.

## Introduction

Jäger (2000) proposed the huntsman spider genus *Pseudopoda* by re-describing *P. prompta* (O. Pickard-Cambridge, 1885) from Pakistan and India. Since then, no fewer than 140 species have been assigned to this genus, which is now known to occur in areas from South, East and Southeast Asia. Of this diversity, 61 species have been recorded from China (World Spider Catalog 2019). Known species are mainly collected in the leaf litter, underneath tree bark, under stones and on plants (Jäger and Vedel 2007).

**Figure 1.**
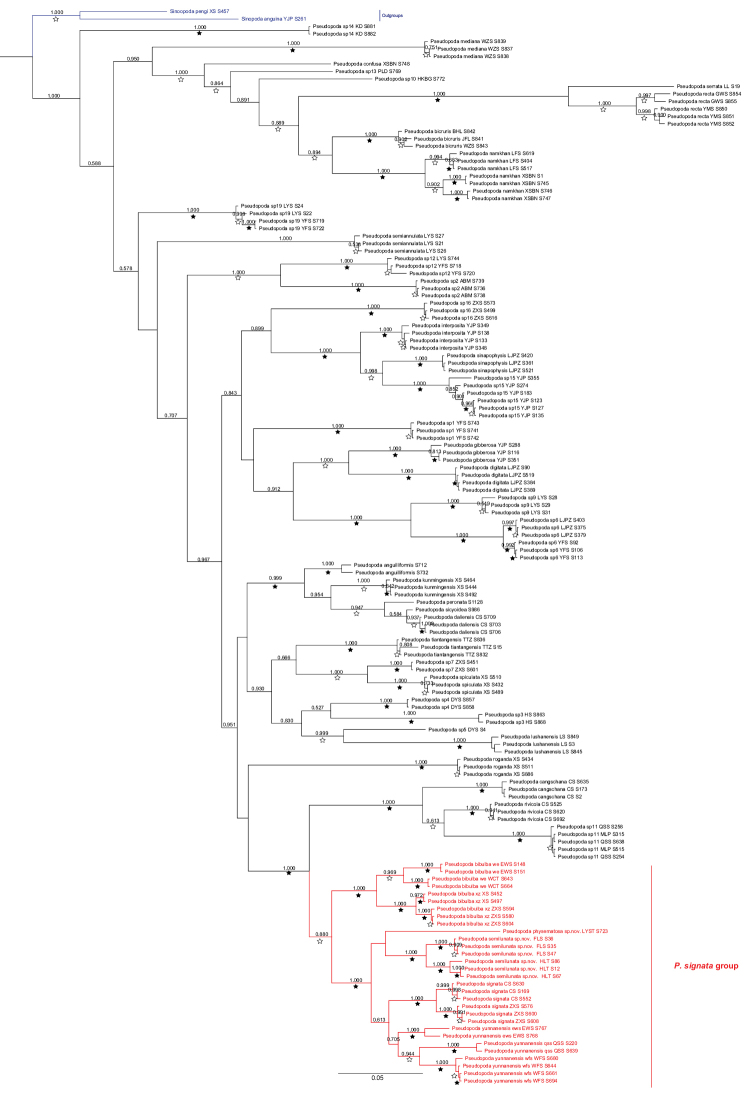
Bayesian tree based on the COI + ITS2 dataset including 144 *Pseudopoda* individuals belonging to 44 species. Numbers on nodes are posterior probabilities; bootstrap support from ML analyses is indicated as solid stars for values > 95%, open stars > 50–95%. Red clade indicates the *P. signata* group, blue clade indicates the outgroups. Phylogenetic tree cited from Zhang et al. (2017).

Jäger (2001) established six species groups within the genus according to morphological evidences based on species mostly collected from Himalayas and nearby mountain ranges: *P. diversipunctata* group, *P. latembola* group, *P. martensi* group, *P. parvipunctata* group, *P. prompta* group, and *P. schwendingeri* group. Nevertheless, the monophyly of these groups has never been tested by any phylogenetical analysis. Cao et al. (2016) published a molecular phylogeny on Chinese *Pseudopoda* species based on COI and ITS2 genes data, focusing on DNA barcoding of this genus, without discussing species groups. Zhang et al. (2017) established the seventh *Pseudopoda* group (*P. daliensis* group including five species from Yunnan Province, China) based on morphological and molecular data which are mostly cited from Cao et al. (2016). So far, only 47 (33.57%) species were assigned to species groups, since it is challenging to group species exclusively according to morphological data of a limited set of species. Jäger (2001) described *P. signata* but did not assign it to any species group considering the female a transitional form between the *prompta* group and the *martensi* group. Here, we expand the baseline data for such decision by evaluating molecular (Fig. 1, edited from Zhang et al. 2017: fig. 1) as well as morphological evidence (see taxonomy), and establish the *P. signata* group, to which we assign seven species, two of which new to science, from Guizhou, Sichuan, and Yunnan provinces in China.

## Material and methods

All specimens were preserved in 75% ethanol and examined with an Olympus SZX16 stereomicroscope; details were further investigated with an Olympus BX51 compound microscope. Male and female copulatory organs were examined and illustrated after dissection from the spider bodies, vulvae were cleared with Proteinase K. Habitus photos were obtained using a Leica 205C digital microscope.

Leg measurements are shown as: total length (femur, patella, tibia, metatarsus, tarsus). The numbers of spines are listed for each segment in the following order: prolateral, dorsal, retrolateral, ventral (in femora and patellae, ventral spines are absent, and the fourth digit is omitted in the spination formula). The body size classes and illustration of schematic course of internal duct system follow Jäger (2001). The terminology used in the text and figure legends follows Quan et al. (2014). All measurements are in millimetres.

We evaluated the most recent phylogenetic evidence for relationships among various *Pseudopoda* species (Fig. 1, edited from Zhang et al. 2017: fig. 1). For detailed phylogenetic methods and abbreviations see Cao et al. (2016) and Zhang et al. (2017).

Abbreviations used throughout the text are given below.

Somatic morphology:

**ALE** anterior lateral eyes;

**AME** anterior median eyes;

**CH** clypeus height;

**CO** copulatory opening;

**dRTA** dorsal part/branch of RTA;

**DS** dorsal shield of prosoma;

**E** embolus;

**Fe** femur;

**FD** fertilisation duct;

**FW** first winding;

**Mt** metatarsus;

**OS** opisthosoma;

**Pa** patella;

**PLE** posterior lateral eyes;

**PME** posterior median eyes;

**Pp** palp;

**RTA** retrolateral tibial apophysis;

**SIDS** sclerotised internal duct system;

**ST** subtegulum;

**T** tegulum;

**Ti** tibia;

**I**, **II**, **III**, **IV** – legs I to IV;

**vRTA** ventral part/branch of RTA.

Institutes:

**CBEE** Centre for Behavioural Ecology and Evolution, College of Life Sciences, Hubei University, Wuhan, China;

**HUST** School of Nuclear Technology and Chemistry & Biology, Hubei University of Science and Technology, Xianning, Hubei, China;

**SWUC** College of Life Sciences, Southwest University, Chongqing, China.

## Taxonomy

### Family Sparassidae Bertkau, 1872

#### Subfamily Heteropodinae Thorell, 1873

##### Genus *Pseudopoda* Jäger, 2000

###### *Pseudopoda signata* group

**Figure 2.**
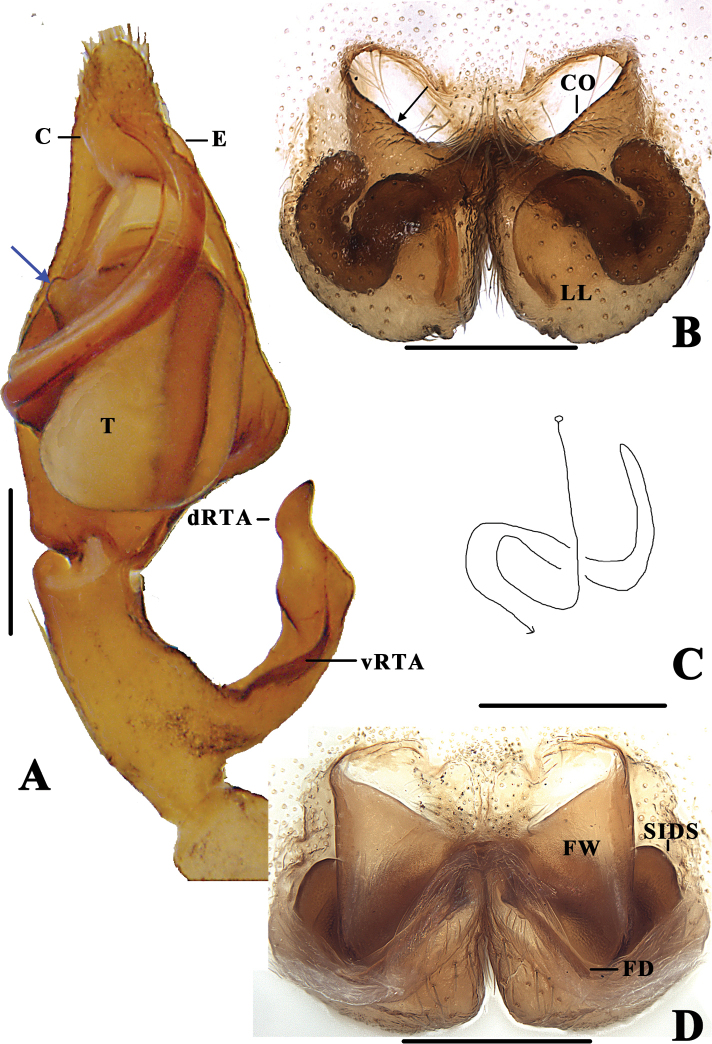
*Pseudopoda bibulba* Xu & Yin, 2000 **A** left male palp, ventral **B** epigyne, ventral **C** schematic course of internal duct system in right part, dorsal **D** vulva, dorsal. Black arrow pointing to anterior margin of lateral lobe, blue arrow to tegular outgrowth. Abbreviations: C–conductor; dRTA–dorsal retrolateral tibial apophysis; E–embolus; vRTA–ventral retrolateral tibial apophysis; T–tegulum; CO–copulatory opening; LL–lateral lobes; FD–fertilisation duct; FW–first winding; SIDS–sclerotised internal duct system. Scale bars: 0.5 mm.

**Definition.** This group can be recognised by the combination of the following characters:

1. Embolus distinctly longer than tegulum, slightly S-shaped, arising from tegulum between 7- AND 9-o’clock-position (Fig. 2A);

2. Tegulum with distinctly short tegular outgrowth (Fig. 2A);

3. dRTA pronounced, vRTA short, dRTA two times longer than vRTA (Fig. 2A);

4. Anterior margins of lateral lobes bent medially, roughly “U” or “V”-shaped (Fig. 2B);

5. First winding membranous and wide, covering large part of the sclerotised internal duct system (Fig. 2D);

6. Sclerotised part of internal duct system long, strongly curved, tube-shaped (Fig. 2D).

**Composition.***P. bibulba* Xu & Yin, 2000, *P. physematosa* sp. nov., *P. semilunata* sp. nov., *P. signata* Jäger, 2001, *P. wu* Jäger, Li & Krehenwinkel, 2015, *P. yinae* Jäger & Vedel, 2007, *P. yunnanensis* Yang & Hu, 2001.

**Distribution.** China (Guizhou, Sichuan, Yunnan provinces) (Fig. 18).

####### Pseudopoda bibulba

Taxon classificationAnimaliaAraneaeSparassidae

Xu & Yin, 2000

0AB1B836-070B-5225-AE3B-7EA23BE9252A

Figs 2 , 3 , 4 , 18

Heteropoda bibulba Xu & Yin, 2000: 37, figs 1–3 (description of female).
Pseudopoda bibulba : Jäger & Yin, 2001: 126 (transfer from Heteropoda); Jäger & Vedel, 2007: 15, figs 44–59 (description of male, redescription of female).

######## Material examined.

**CHINA, *Guizhou Province***: 3 females, Liupanshui City, Zhongshan District, Xianshui slope martyr cemetery, 26.61°N, 104.84°E, 1966 m, 11 April 2016, Yang Zhong, Yang Zhu & He Zhang leg. (CBEE, LJ02358-LJ02360); ***Yunnan Province***: 19 males, 14 females, Kunming City, Xishan Scenic Area, 24.96°N, 102.63°E, 1975 m, 14 May 2014, Yang Zhong & Xiaowei Cao leg. (CBEE, LJ02361-LJ02393); 1 female, Kunming City, Xishan Scenic Area, 24.96°N, 102.63°E, 2204 m, 13 October 2016, Guiqiang Huang, Xiangbo Guo and Yanchao Wang leg. (CBEE, LJ02394); 19 males, 14 females, Chuxiong City, Zixishan Scenic Area, 25.01°N, 101.42°E, 2527 m, 15 May 2014, Yang Zhong & Xiaowei Cao leg. (CBEE, LJ02395-LJ02427); 5 males, 3 females, Nujiang Lisu Autonomous Prefecture, Lanping Bai Nationality Autonomous Prefecture, Mt. Erwu, 26.43°N, 99.41°E, 2377 m, 28 May 2014, Yang Zhong & Xiaowei Cao leg. (CBEE, LJ02428-LJ02435); 6 males, 5 females, Wei Xi Lisu Autonomous County, Pagoda of Cultural Prosperity, 27.18°N, 99.29°E, 2294 m, 26 May 2014, Yang Zhong & Xiaowei Cao leg. (CBEE, LJ02436-LJ02446).

**Figure 3.**
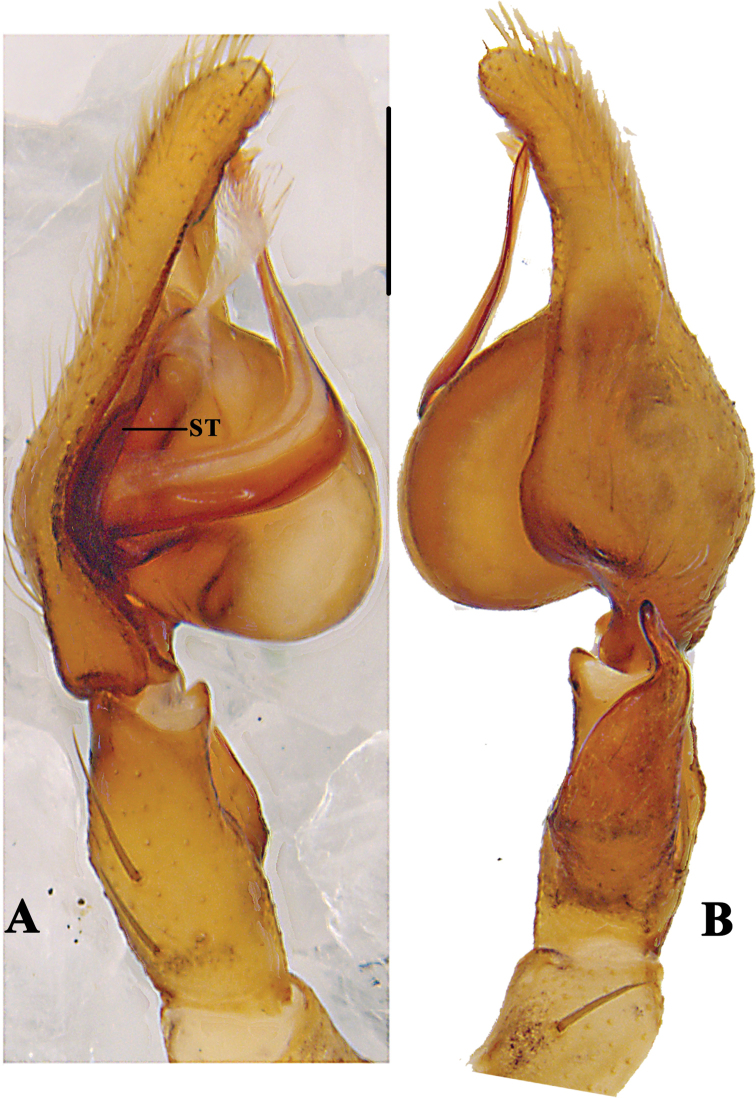
*Pseudopoda bibulba* Xu & Yin, 2000 **A** left male palp, prolateral **B** same, retrolateral. Abbreviation: ST–subtegulum. Scale bar: 0.5 mm.

**Figure 4.**
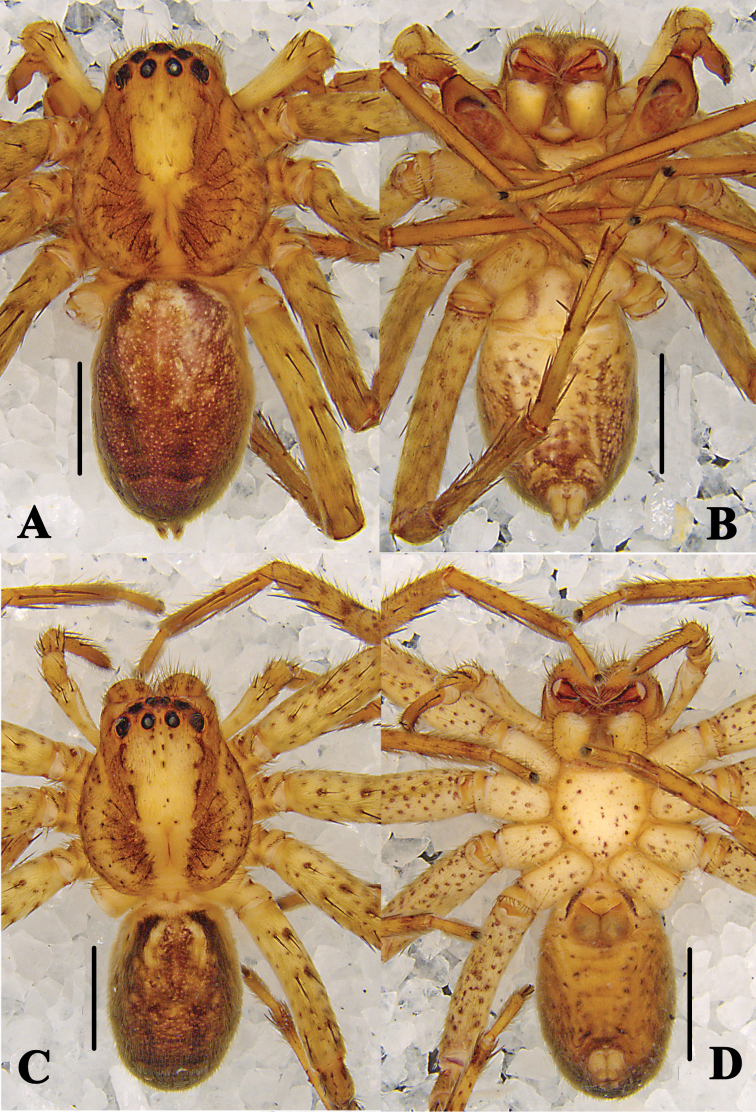
*Pseudopoda bibulba* Xu & Yin, 2000 **A, B** male habitus (**A** dorsal **B** ventral) **C, D** female habitus (**C** dorsal **D** ventral). Scale bars: 2 mm.

######## Diagnosis and Description.

See Jäger and Vedel (2007).

######## Distribution.

China (Guizhou, new province record; Yunnan) (Fig. 18).

####### Pseudopoda physematosa sp. nov.

Taxon classificationAnimaliaAraneaeSparassidae

A9624771-823A-515F-B49F-732E8C90AF98

http://zoobank.org/8AD4A005-5F68-438F-997A-32F410F14B7A

Figs 5 , 6 , 7 , 18

######## Type material.

**Holotype female: CHINA: *Yunnan Province***: Lijiang City, Yongsheng County, Lingyuan Temple, 26.70°N, 100.78°E, 2305 m, 25 August 2013, Yang Zhong & Xiaowei Cao leg. (CBEE, LJ01667); **Paratypes**: 2 females, with same data as holotype. (CBEE, LJ01668-LJ01669).

######## Etymology.

**Figure 5.**
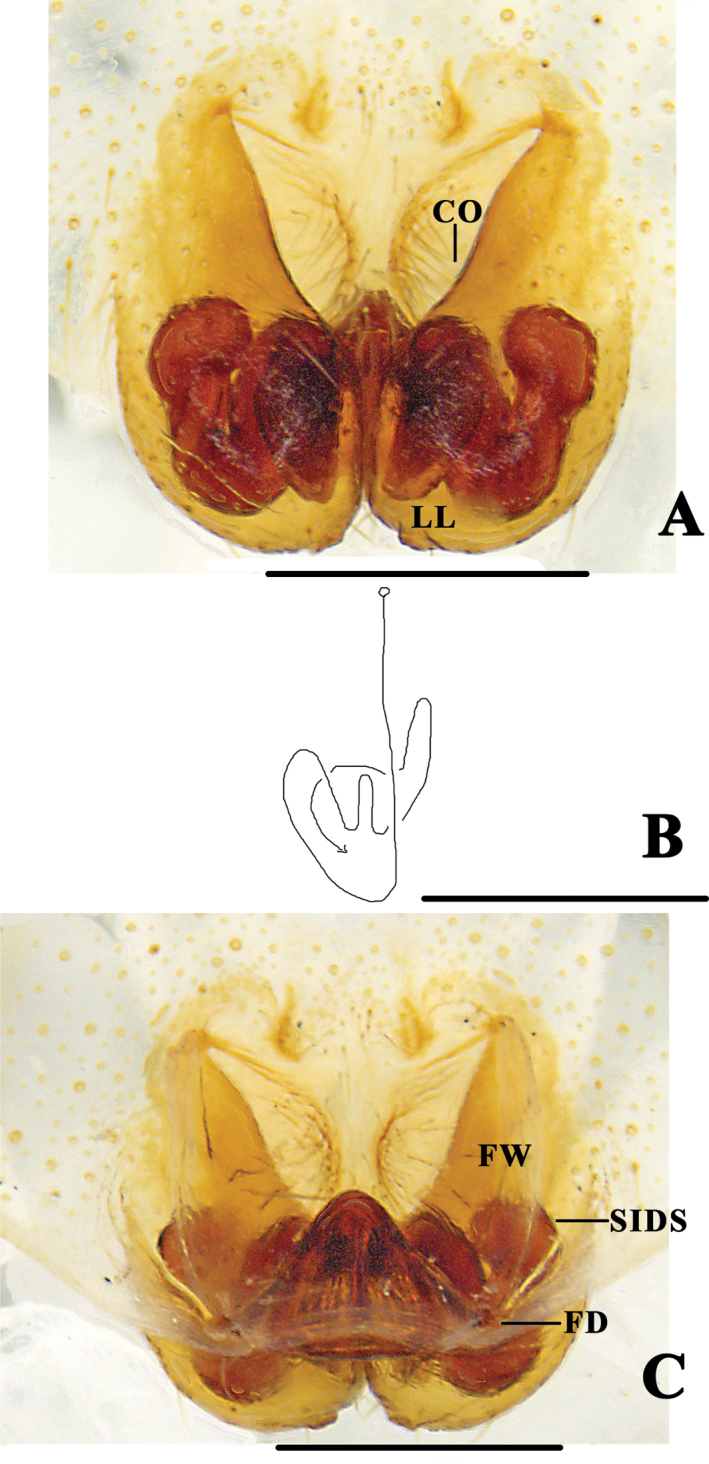
*Pseudopoda physematosa* sp. nov. **A** epigyne, ventral **B** schematic course of internal duct system, dorsal **C** vulva, dorsal. Abbreviations: CO–copulatory opening; FD–fertilisation duct; FW–first winding; LL–lateral lobes; SIDS–sclerotised internal duct system. Scale bars: 0.5 mm.

The specific name is derived from the Latin adjective *physematosus*, -*a*, -*um*, meaning swollen, referring to the shape of SIDS in dorsal view (Fig. 5C); adjective.

######## Diagnosis.

*Pseudopoda physematosa* sp. nov. differs from other species of the *P. signata* group, except *P. bibulba*, by the SIDS with well developed twisted loops, in ventral view. It can be distinguished from *P. bibulba* by the following characters: anterior margins of lateral lobes longer than half the width of epigynal field in *P. physematosa*, but shorter in *P. bibulba*; SIDS folded, with swollen tip in *P. physematosa*, but not in *P. bibulba* (Fig. 5A).

######## Description.

**Male** unknown. **Female (holotype)**: Measurements: small-sized Heteropodinae. Body length 8.6–9.9. DS length 4.4, width 3.3, OS length 5.5, width 3.6. Eyes: AME 0.24, ALE 0.31, PME 0.18, PLE 0.22, AME-AME 0.18, AME-ALE 0.11, PME-PME 0.15, PME-PLE 0.24, AME-PME 0.27, ALE-PLE 0.24, CHAME 0.35, CHALE 0.28. Leg formula: II-I-IV-III. Spination: Pp 131, 101, 2121, 1014; Fe I–II 323, III 322, IV 322; Pa I–III 101, IV 100; Ti I–II 2228, III–IV 2126; Mt I–II 2024, III 3025, IV 3036. Measurement of palps and legs: Pp 4.5 (1.3, 0.5, 1.0, -, 1.7); I 12.8 (3.8, 1.9, 2.8, 3.1, 1.2); II 13.0 (4.0, 1.3, 3.0, 3.2, 1.5); III 11.1 (3.1, 1.6, 2.7, 2.6, 1.1); IV 11.6 (3.1, 1.7, 3.0, 2.7, 1.1). Promargin of chelicerae with three teeth, retromargin with four teeth, cheliceral furrow with ca. 32 denticles. Epigynal field almost as wide as long, with anterior bands included in the field. Epigyne with lateral lobes touching each other posteriorly. The anterior margins of lateral lobes forming a “V”. FW covering most of SIDS, the latter folded in the middle part (Fig. 5A–C). Colouration in ethanol: DS yellow with irregular radially arranged dark spots and brown patterns. Fovea and radial furrows distinctly marked. OS dorsally with light yellow hairs and large patches of reddish brown spots, ventrally lighter with larger and sparser reddish brown marks (Fig. 7A, B).

**Figure 6.**
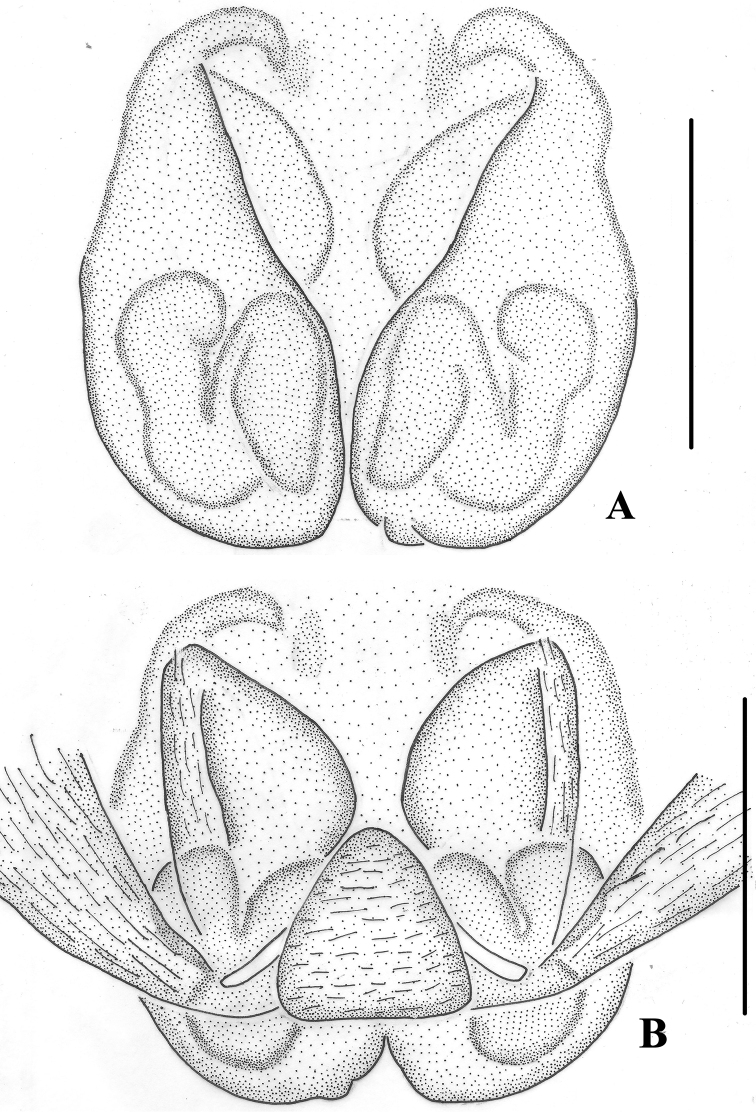
*Pseudopoda physematosa* sp. nov. **A** epigyne, ventral **B** vulva, dorsal. Scale bars: 0.5 mm.

**Figure 7.**
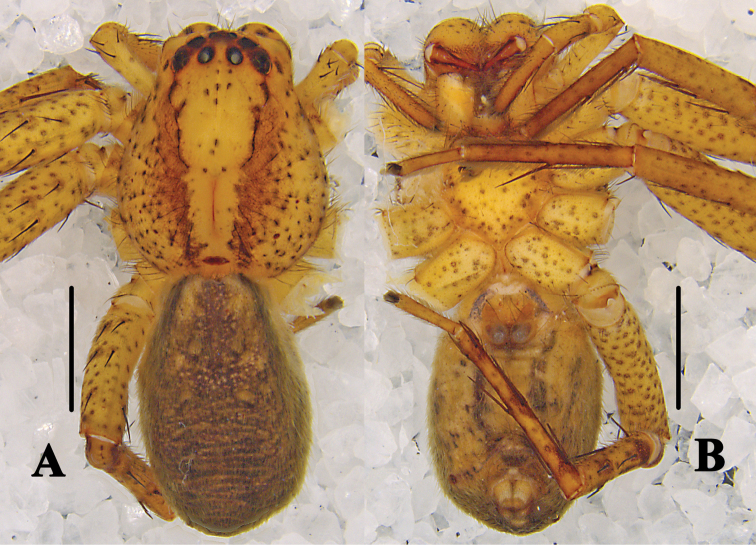
*Pseudopoda physematosa* sp. nov. Female habitus (**A** dorsal **B** ventral). Scale bars: 2 mm.

######## Distribution.

China (Yunnan) (Fig. 18).

####### Pseudopoda semilunata sp. nov.

Taxon classificationAnimaliaAraneaeSparassidae

BF502C6E-3259-5955-A252-FE7C3BF80A8E

http://zoobank.org/42780378-AF52-41DB-8447-C71F62523204

**Figure 8.**
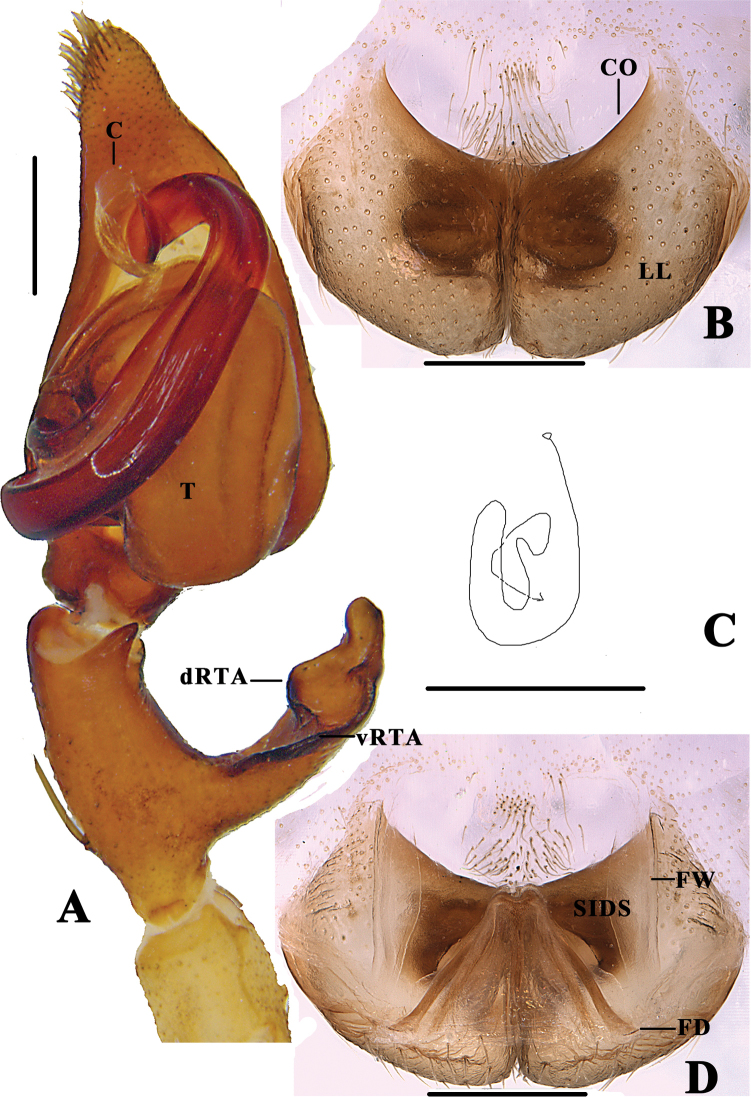
*Pseudopoda semilunata* sp. nov. **A** left male palp, ventral **B** epigyne, ventral **C** schematic course of internal duct system in right art, dorsal **D** vulva, dorsal. Abbreviations: C–conductor; dRTA–dorsal retrolateral tibial apophysis; T–tegulum; vRTA–ventral retrolateral tibial apophysis; CO–copulatory opening; LL–lateral lobes; FD–fertilisation duct; FW–first winding; SIDS–sclerotised internal duct system. Scale bars: 0.5 mm.

**Figure 9.**
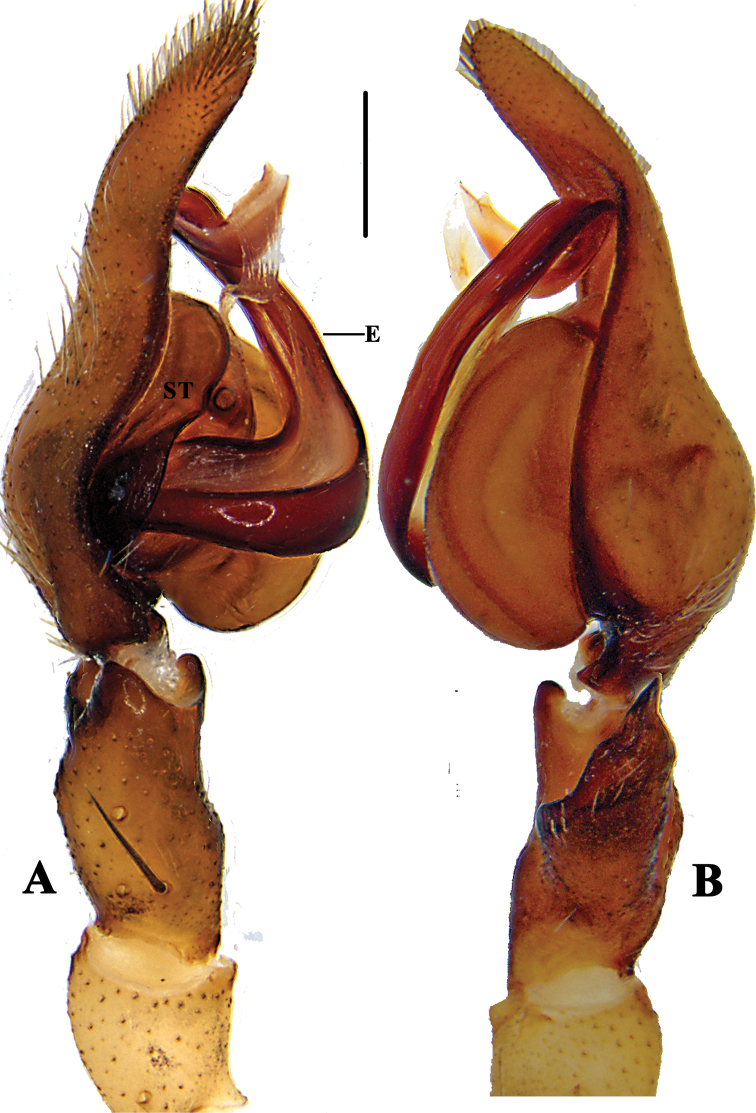
*Pseudopoda semilunata* sp. nov. **A** left male palp, prolateral **B** Same, retrolateral. Abbreviations: E-embolus; ST-subtegulum. Scale bar: 0.5 mm.

**Figure 10.**
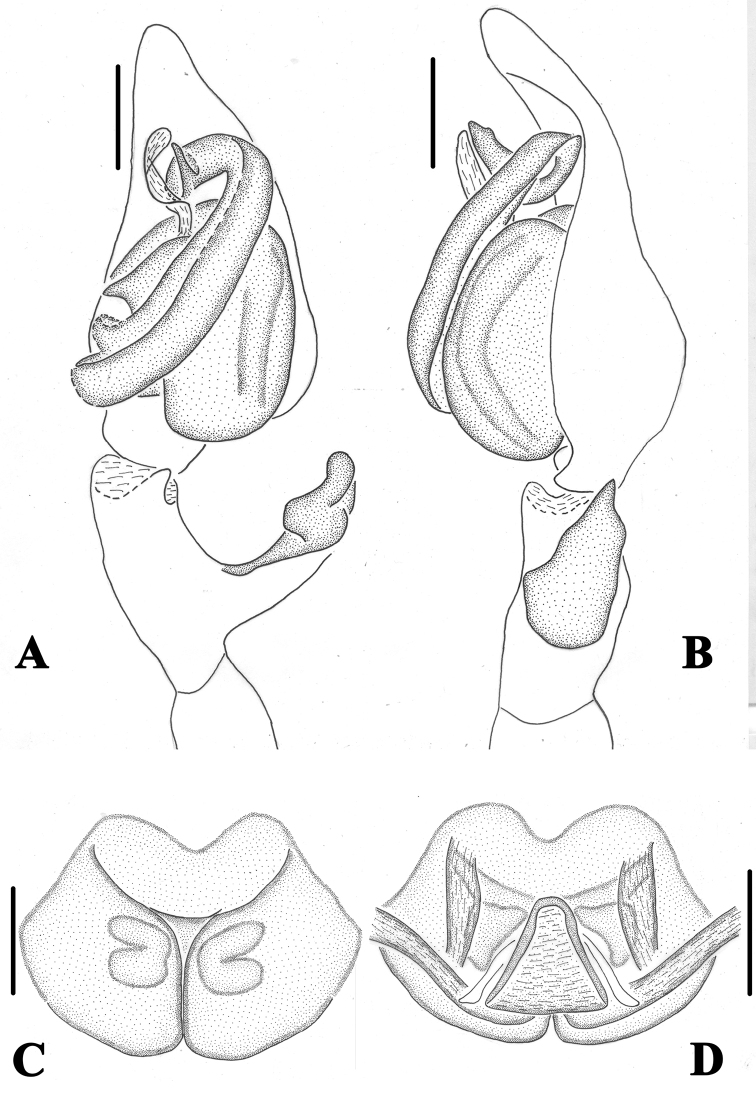
*Pseudopoda semilunata* sp. nov. **A, B** left male palp (**A** ventral **B** retrolateral) **C** epigyne, ventral **D** vulva, dorsal. Scale bars: 0.5mm.

**Figure 11.**
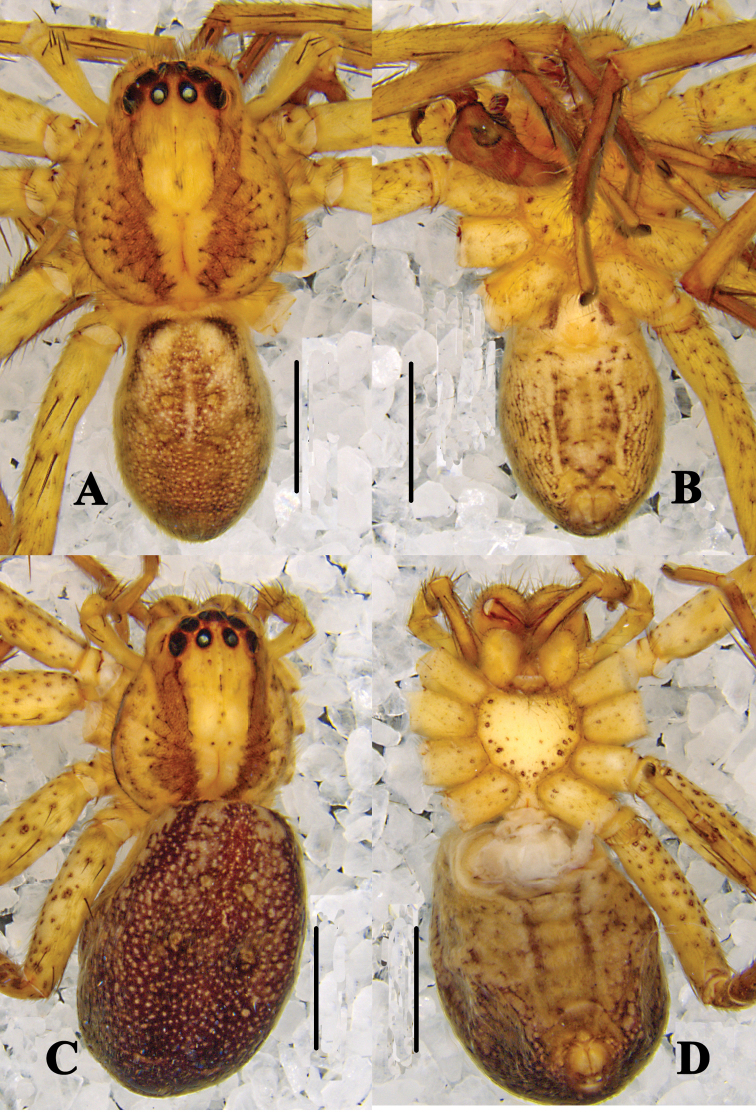
*Pseudopoda semilunata* sp. nov. **A, B** male habitus (**A** dorsal **B** ventral) **C, D** female habitus (**C** dorsal **D** ventral). Scale bars: 2 mm.

Figs 8 , 9 , 10 , 11 , 18

######## Type material.

**Holotype male: CHINA: *Yunnan Province***: Lijiang City, Black Dragon Pool Park, 26.89°N, 100.24°E, 2659 m, 20 May 2014, Yang Zhong & Xiaowei Cao leg. (CBEE, LJ01905); **Paratypes**: 7 males, 8 females, with same data as holotype. (CBEE, LJ01906-LJ01920); 2 males, 2 females, Diqing Tibetan Autonomous Prefecture, Deqin County, Fei Lai Temple scenic area, 28.42°N, 98.87°E, 3458m, 25 May 2014, Yang Zhong & Xiaowei Cao leg. (CBEE, LJ01921-LJ01924).

######## Etymology.

The specific name is derived from the Latin adjective *semilunatus*, -*a*, -*um*, meaning lunate, referring to the shape of anterior margins of lateral lobes (Fig. 8B–D); adjective.

######## Diagnosis.

*P. semilunata* sp. nov. differs from other members in this group by the following characters: dRTA with distinct sub-apical cavity, anterior margins of lateral lobes not strongly curved as in other species but together forming a semicircle. Males of this species are similar to those of *P. wu* in having a twisted embolus tip but can be distinguished by embolic tip forming a semicircle and conductor present (embolic tip forming a full circle, conductor entirely reduced in *P. wu*) (Figs 8A–D, 9A, B).

######## Description.

**Male (holotype)**: Measurements: small-sized Heteropodinae. Body length 7.0–9.5. DS length 4.1, width 3.5, OS length 5.0, width 3.4. Eyes: AME 0.16, ALE 0.23, PME 0.19, PLE 0.24, AME-AME 0.13, AME-ALE 0.21, PME-PME 0.24, PME-PLE 0.27, AME-PME 0.37, ALE-PLE 0.40, CHAME 0.26, CHALE 0.21. Leg formula: II-I-IV-III. Spination: Pp 131, 101, 2121, 1014; Fe I–II 323, III 322, IV 322; Pa I–III 101, IV 100; Ti I–II 2228, III–IV 2126; Mt I–II 2024, III 3025, IV 3036. Measurement of palps and legs: Pp 5.0 (1.3, 0.6, 0.9, -, 2.2); I 13.9 (3.9, 1.0, 3.6, 4.0, 1.4); II 14.3 (4.1, 1.2, 4.2, 3.3, 1.5); III 12.2 (3.5, 0.9, 3.4,3.0, 1.4); IV 13.7 (4.5, 1.2, 3.3, 3.2, 1.5). Promargin of chelicerae with three teeth, retromargin with four teeth, cheliceral furrow with ca. 20 denticles. Palp as in diagnosis. Conductor arising from tegulum at 12-o’clock-position, basally folded. Tegular outgrowth short, claviform. Embolus arising from tegulum at 8.30-o’clock-position, long, well developed, with abruptly tapering apical part with additional loop. Spermophore visible submarginally on retrolateral tegulum in ventral view. RTA arising proximally on Ti, with broad ventral part, its distal end bent, bowl-shaped (Figs 8A, 9A, B). Colouration in ethanol: DS yellow with dark spots, two lateral bands, margin with thin dash line and brown patterns. Fovea and radial furrows distinctly marked. OS dorsally with lots of reddish brown dots, ventrally with reddish brown marks, regularly arranged (Fig. 11A, B).

**Female**: Measurements: small-sized Heteropodinae. Body length 8.0–9.5. DS length 4.0, width 3.6, OS length 4.6, width 2.8. Eyes: AME 0.15, ALE 0.20, PME 0.16, PLE 0.20, AME-AME 0.27, AME-ALE 0.13, PME-PME 0.33, PME-PLE 0.41, AME-PME 0.35, ALE-PLE 0.32, CHAME 0.31, CHALE 0.29. Leg formula: II-I-IV-III. Spination: Pp 131, 101, 2121, 1014; Fe I–II 323, III 322, IV 322; Pa I–III 101, IV 100; Ti I–II 2228, III–IV 2126; Mt I–II 2024, III 3025, IV 3036. Measurements of palps and legs: Pp 4.4 (1.3, 0.6, 0.9, -, 1.6); I 9.9 (3.1, 0.8, 2.6, 2.4, 1.0); II 10.9 (3.1, 1.2, 3.0, 2.6, 1.0); III 7.3 (1.9, 0.8, 2.2, 1.5, 0.9); IV 8.8 (2.8, 0.8, 2.4, 2.0, 0.8). Cheliceral furrow with three anterior and four posterior teeth, and with ca. 18 denticles. Epigynal field wider than long. Anterior and posterior margins of lateral lobes almost parallel. FW well developed, covering the entire sclerotised part of internal duct system, the latter folded. FD long, narrow (Fig. 8B–D). Colouration in ethanol: As in male, generally darker (Fig. 11C, D).

######## Distribution.

China (Yunnan) (Fig. 18).

####### Pseudopoda signata

Taxon classificationAnimaliaAraneaeSparassidae

Jäger, 2001

93223859-E006-57B9-952B-FDDA54B9DA6A

**Figure 12.**
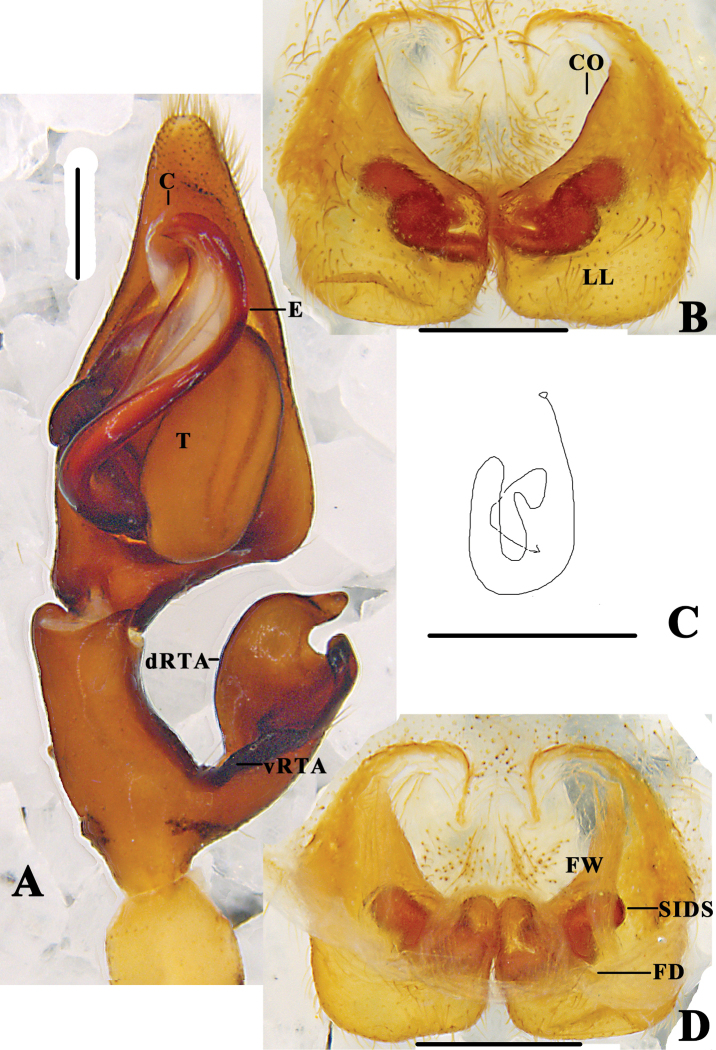
*Pseudopoda signata* Jäger, 2001 **A** left male palp, ventral **B** epigyne, ventral **C** schematic course of internal duct system in right part, dorsal **D** vulva, dorsal. Abbreviations: C–conductor; E–embolus; dRTA–dorsal retrolateral tibial apophysis; T–tegulum; vRTA–ventral retrolateral tibial apophysis; CO–copulatory opening; LL–lateral lobes; FD–fertilisation duct; FW–first winding; SIDS–sclerotised internal duct system. Scale bars: 0.5 mm.

**Figure 13.**
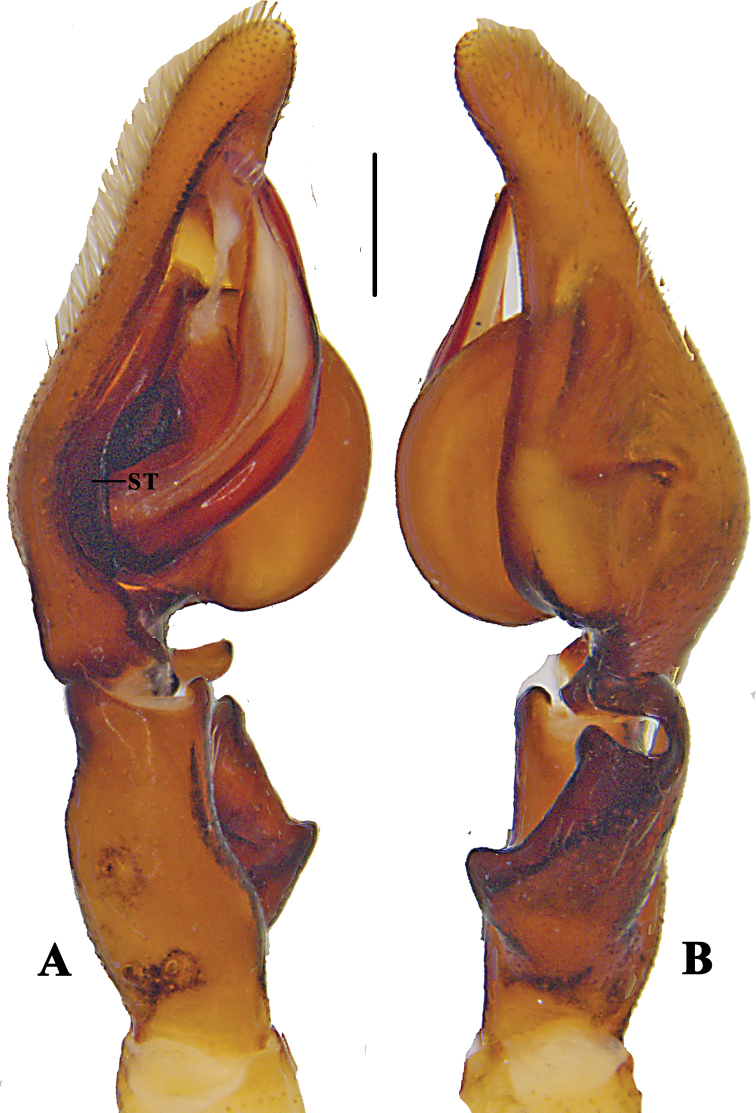
*Pseudopoda signata* Jäger, 2001 **A** left male palp, prolateral **B** same, retrolateral. Abbreviation: ST–subtegulum. Scale bar: 0.5 mm.

**Figure 14.**
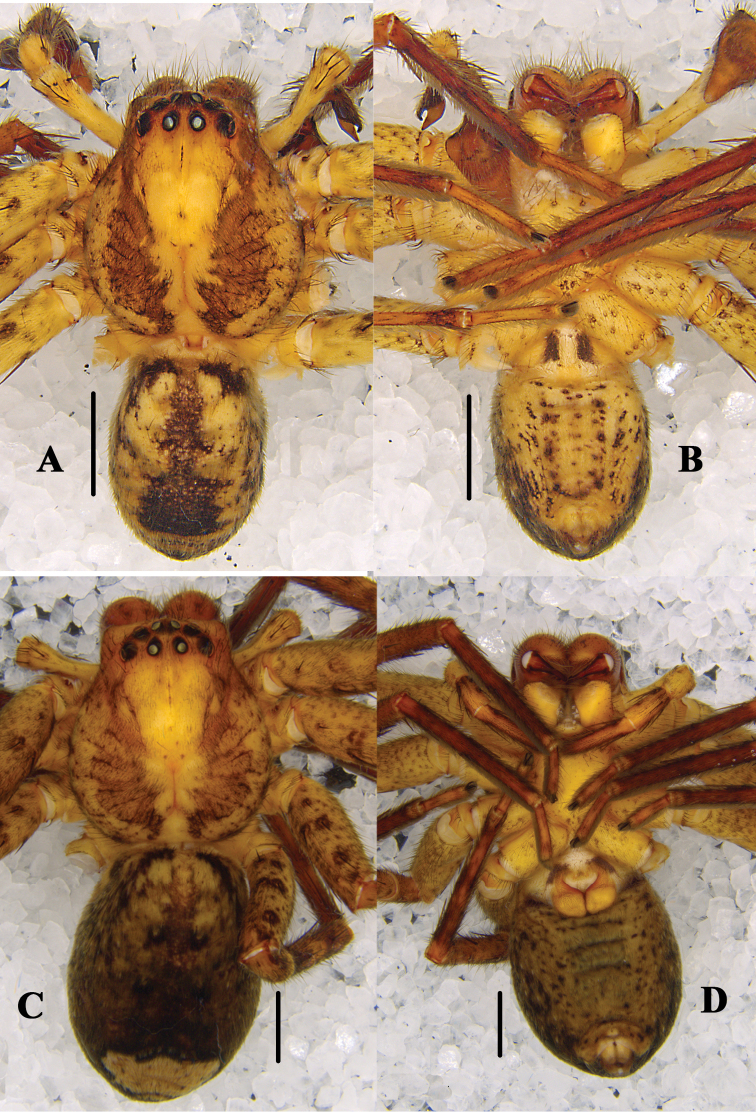
*Pseudopoda signata* Jäger, 2001 **A, B** male habitus (**A** dorsal **B** ventral) **C, D** female habitus (**C** dorsal **D** ventral). Scale bars: 2 mm

Figs 12 , 13 , 14 , 18

Pseudopoda signata Jäger, 2001: 50, f. 29h-j (description of female).
Pseudopoda signata : Jäger et al. 2015: 375, f, 55–90, 93–106 (description of male, redescription of female); Jäger, 2015: 349, fig. 98 (illustration of male).

######## Material examined.

**CHINA, *Yunnan Province***: 6 males, 7 females, Dali Bai Autonomous Prefecture, Cangshan Scenic Area, 25.01°N, 100.14°E, 2645 m, 17 May 2014, Yang Zhong & Xiaowei Cao leg. (CBEE, LJ01695-LJ01707); 12 males, 6 females, Chuxiong City, Zixishan Scenic Area, 25.01°N, 101.42°E, 2476 m, 15 May 2014, Yang Zhong & Xiaowei Cao leg. (CBEE, LJ01708-LJ01719, LJ01785-LJ01790).

######## Diagnosis and Description.

See Jäger et al. (2015).

######## Distribution.

China (Yunnan, new province record; Sichuan) (Fig. 18).

####### Pseudopoda yunnanensis

Taxon classificationAnimaliaAraneaeSparassidae

Yang & Hu, 2001

81A7F39C-E403-579F-AFD4-BF4A9D992670

**Figure 15.**
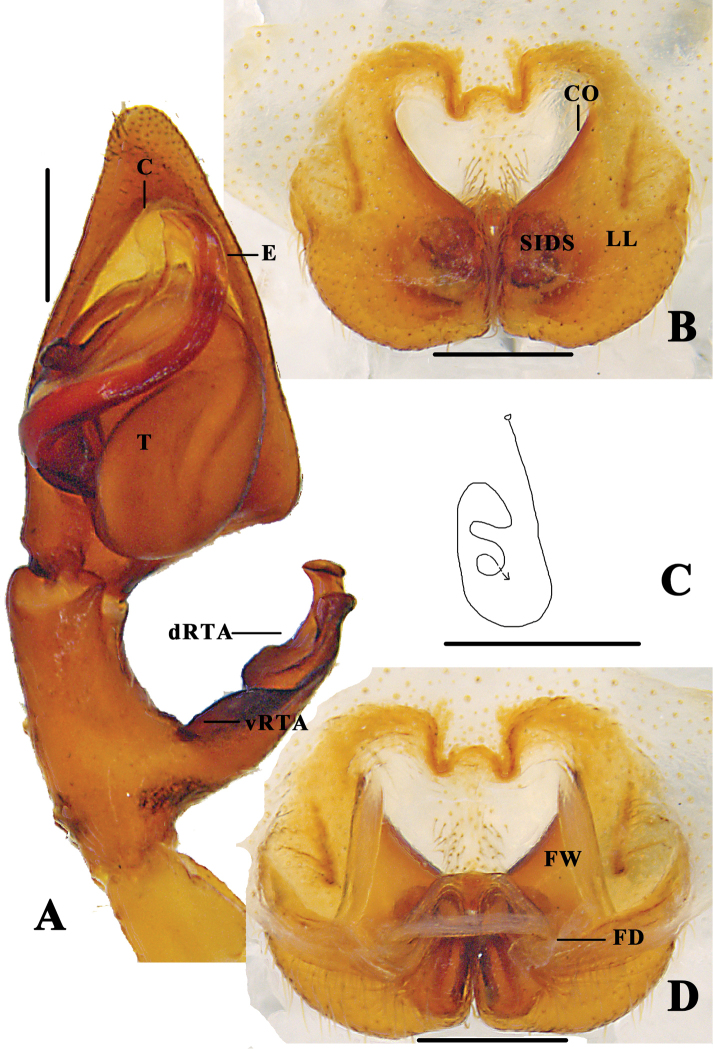
*Pseudopoda yunnanensis* Yang & Hu, 2001 **A** left male palp, ventral **B** epigyne, ventral **C** Schematic course of internal duct system in right part, dorsal **D** vulva, dorsal. Abbreviations: C–conductor; dRTA–dorsal retrolateral tibial apophysis; E–embolus; vRTA–ventral retrolateral tibial apophysis; T–tegulum; CO–copulatory opening; LL–lateral lobes; FD–fertilisation duct; FW–first winding; SIDS–sclerotised internal duct system. Scale bars: 0.5 mm.

**Figure 16.**
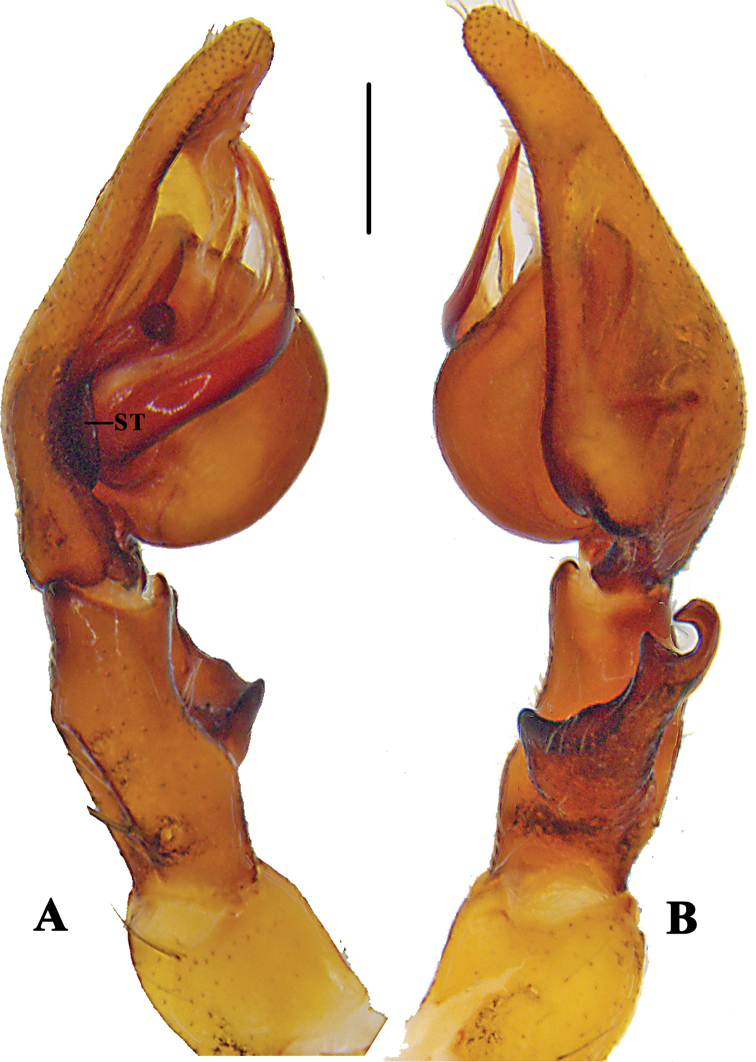
*Pseudopoda yunnanensis* Yang & Hu, 2001 **A** left male palp, prolateral **B** same, retrolateral. Abbreviation: ST–subtegulum. Scale bar: 0.5mm.

**Figure 17.**
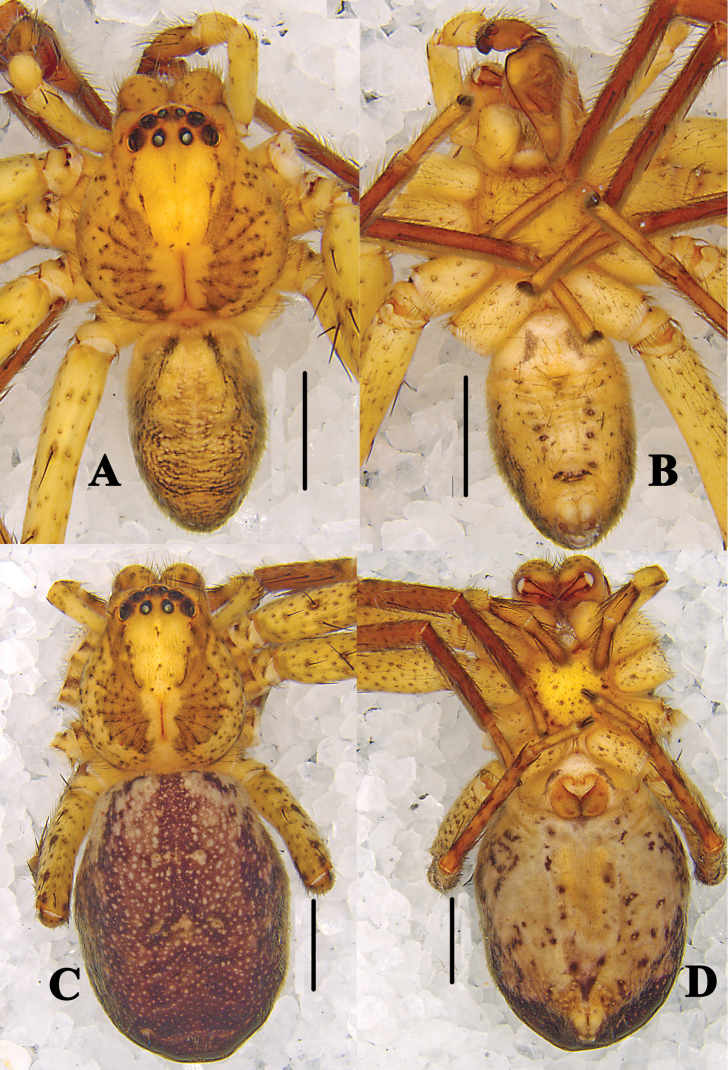
*Pseudopoda yunnanensis* Yang & Hu, 2001 **A, B** male habitus (**A** dorsal **B** ventral) **C, D** female habitus (**C** dorsal **D** ventral). Scale bars: 2 mm

**Figure 18.**
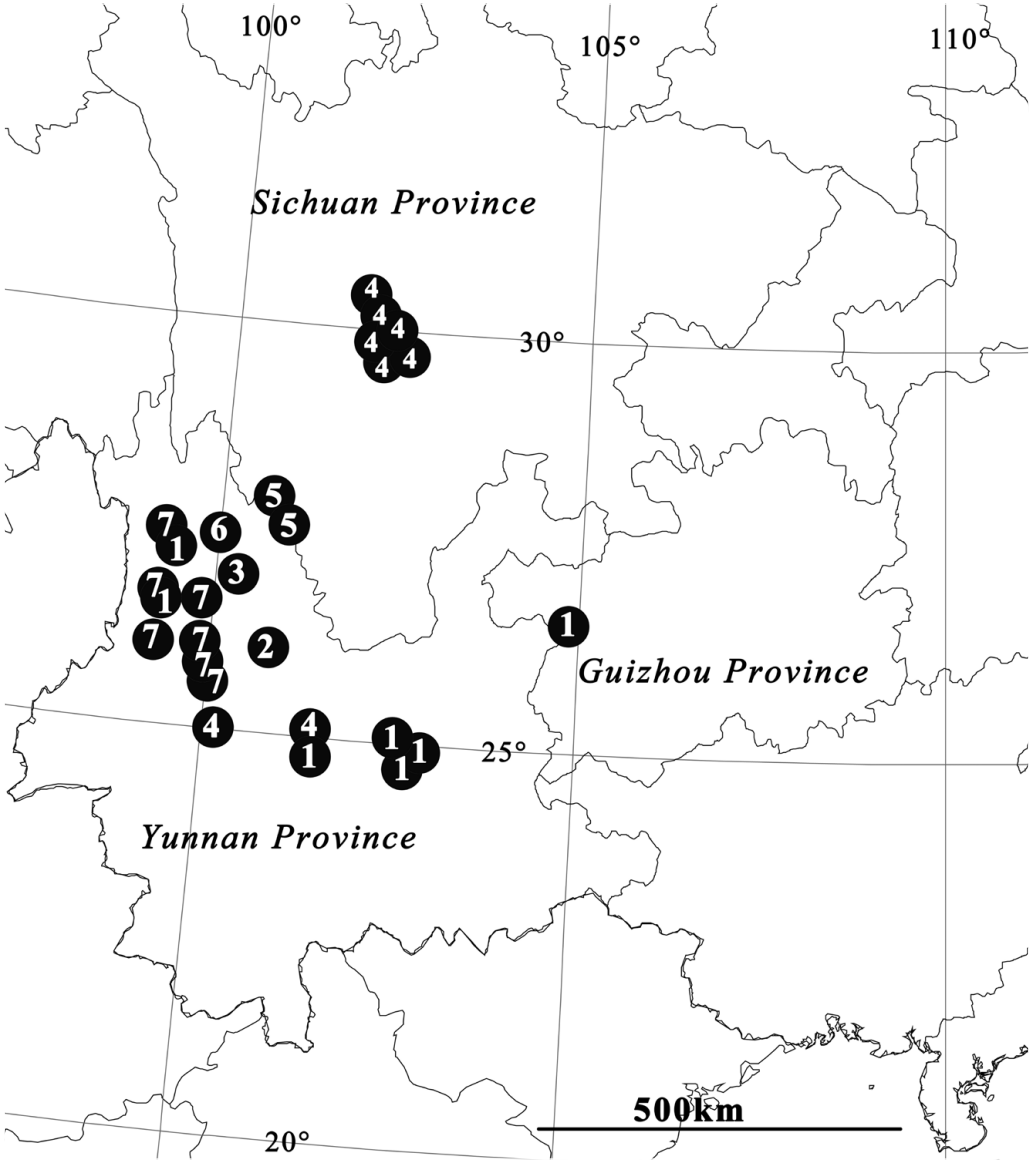
Locality records of *P. signata* group from China: **1***P. bibulba***2***P. physematosa* sp. nov. **3***P. semilunata* sp. nov. **4***P. signata***5***P. wu***6***P. yinae***7***P. yunnanensis*.

Figs 15 , 16 , 17 , 18

Sinopoda yunnanensis Yang & Hu, 2001: 18, figs 1–3 (description of female).
Pseudopoda yunnanensis : Jäger & Vedel, 2007: 17, figs 60–62 (Transfer from Sinopoda); Yang & Chen, 2008: 810, figs 1–13 (Description of male, redescription female).

######## Material examined.

**CHINA, *Yunnan Province***: 2 males, 2 females, Nujiang Lisu Autonomous Prefecture, Lanping Bai Nationality Autonomous Prefecture, Mt. Erwu, 26.43°N, 99.41°E, 2366 m, 28 May 2014, Yang Zhong & Xiaowei Cao leg. (CBEE, LJ01535-LJ01538); 42 males, 26 females, Diqing Tibetan Autonomous Prefecture, Shangri-La County, Mt. Wufeng, 27.18°N, 99.29°E, 3528 m, 23 May 2014, Yang Zhong & Xiaowei Cao leg. (CBEE, LJ01539-LJ01606); 18 males, 17 females, Dali Bai Autonomous Prefecture, Jianchuan County, Mt. Qianshi, 26.53°N, 99.88°E, 2647 m, 19 May 2014, Yang Zhong & Xiaowei Cao leg. (CBEE, LJ01501-LJ01534).

######## Diagnosis and Description.

See Yang and Chen (2008).

######## Distribution.

China (Yunnan) (Fig. 18).

## Supplementary Material

XML Treatment for Pseudopoda bibulba

XML Treatment for Pseudopoda physematosa

XML Treatment for Pseudopoda semilunata

XML Treatment for Pseudopoda signata

XML Treatment for Pseudopoda yunnanensis

## References

[B1] Cao XW Liu J Chen J Zheng G Kuntner M Agnarsson I (2016) Rapid dissemination of taxonomic discoveries based on DNA barcoding and morphology. Scientific Reports 6, 37066. 10.1038/srep37066 PMC5171852 27991489

[B2] Jäger P (2000) Two new heteropodine genera from southern continental Asia (Araneae: Sparassidae). Acta Arachnologica 49(1): 61–71. 10.2476/asjaa.49.61

[B3] Jäger P (2001) Diversität der Riesenkrabbenspinnen im Himalaya – die Radiation zweier Gattungen in den Schneetropen (Araneae, Sparassidae, Heteropodinae). Courier Forschungsinstitut Senckenberg 232: 1–136.

[B4] Jäger P (2015) Conductor-less and vertically niched: new species of the genus *Pseudopoda* (Araneae: Sparassidae: Heteropodinae) from Myanmar. Arachnology 16(9): 333–350. 10.13156/arac.2015.16.9.333

[B5] Jäger P Li SQ Krehenwinkel H (2015) Morphological and molecular taxonomic analysis of *Pseudopoda* Jäger, 2000 (Araneae: Sparassidae: Heteropodinae) in Sichuan Province, China. Zootaxa 3999(3): 363–392. 10.11646/zootaxa.3999.3.3 26623582

[B6] Jäger P Vedel V (2007) Sparassidae of China 4. The genus *Pseudopoda* (Araneae: Sparassidae) in Yunnan Province. Zootaxa 1623: 1–38. 10.11646/zootaxa.1623.1.1

[B7] Jäger P Yin C (2001) Sparassidae in China. 1. Revised list of known species with new transfers, new synonymies and type designations (Arachnida: Araneae). Acta Arachnologica 50: 123–134. 10.2476/asjaa.50.123

[B8] Quan D Zhong Y Liu J (2014) Four *Pseudopoda* species (Araneae: Sparassidae) from southern China. Zootaxa 3754(5): 555–571. 10.11646/zootaxa.3754.5.2 24869707

[B9] World Spider Catalog (2019) World Spider Catalog. Natural History Museum Bern. http://wsc.nmbe.ch, version 19.5 [Accessed on August 8, 2018]

[B10] Xu X Yin CM (2000) One new species of the genus *Heteropoda* from China (Araneae: Heteropodidae). Acta Laser Biology Sinica 9: 37–39.

[B11] Yang Z Chen L (2008) The first description of the female *Pseudopoda yunnanensis* (Araneae, Sparassidae). Acta Zootaxonomica Sinica 33: 810–812.

[B12] Yang Z Hu J (2001) A new species of the genus *Sinopoda* from China (Araneae: Heteropodidae). Acta Arachnologica Sinica 10(2): 18–20.

[B13] Zhang H Jäger P Liu J (2017) One new *Pseudopoda* species group (Araneae: Sparassidae)from Yunnan Province, China, with description of three new species. Zootaxa 4318(2): 271–294. 10.11646/zootaxa.4318.2.3

